# High Secretion of Interleukin-6 and Increased MINCLE Receptor Expression Upon Exposure to Mycobacterial Cord Factor Analog Trehalose-6, 6-Dibehenate (TDB) in Patients with Takayasu Arteritis

**DOI:** 10.2174/1874312901812010030

**Published:** 2018-03-28

**Authors:** Nikhil Gupta, Jayakanthan Kabeerdoss, Hindhumathi Mohan, Ruchika Goel, Debashish Danda

**Affiliations:** Department of Clinical Immunology and Rheumatology, Christian Medical College, Vellore, Tamil Nadu, India

**Keywords:** Takayasu Arteritis, Trehalose-6, 6-dibehenate (TDB), MINCLE, Interleukin-6, Mycobacterial cord factor

## Abstract

**Introduction:**

Suspicion on the association between Takayasu Arteritis (TA) and Tubcerculosis (TB) has been in vogue for years. Prevalence of TB in TA is reported to be higher. We aimed to study innate immune responses in patients with TA on exposure to Trehalose-6,6-dibehenate (TDB), a synthetic analogue of Trehalose-6,6-Dimycolate (TDM, also known as mycobacterial cord factor) in comparison with healthy controls.

**Materials and Methods:**

Patients with type V TA, satisfying 1990 ACR criteria, and age and sex matched healthy controls were recruited. PBMCs were cultured with 5µg/ml, 50µg/ml or without any TDB for 48 hours in RPMI medium inside a 5% Co2 incubator. IL-6, TNF-α and IL-17 were measured in cell culture supernatant, which was separated from the cells at the end of the incubation period. Gene expressions of IL-6, IL-8, TNFα, IFN-γ, MINCLE and BCL-10 were quantified in real time PCR using specific primers and SYBR green chemistry.

**Results:**

Twenty two TA patients and 21 healthy controls were recruited. Both patients and controls showed response by secreting IL-6 and TNF-α upon stimulation by TDB. Relative induction (TDB stimulated TA sample / unstimulated control) of IL-6 was significantly higher in TA [31.88(0.74-168)] patients as compared to healthy controls [1.931(0.644-8.21); p<0.002], when co-cultured with 50µg/ml TDB. The expression of MINCLE, the TDB receptor was higher in TA samples than healthy controls upon TDB stimulation.

**Conclusion:**

Stimulation with mycobacterial synthetic analogue led to higher secretion of IL-6 and higher expression of MINCLE in PBMCs of patients with TA as compared to healthy controls.

## 1 INTRODUCTION

Takayasu Arteritis (TA) is a large vessel vasculitis which predominantly affects the aorta and its main branches (1). The role of micro-organisms like *Mycobacterium Tuberculosis* (MTB) has been proposed in the pathogenesis of TA [1, 2].

Association of TA with TB has been studied for many years, but no strong link has been established. In a cohort of 272 cases with TA, Mwipatayi et al. reported active pulmonary tuberculosis in 20% of their patients [3]. In another autopsy series of 10 TA cases, the evidence of active / healed tuberculosis at various sites was found in four of them [4]. However, studies using Tuberculin Skin Test (TST), quantiferon gamma test (QFT-G) and nucleic acid detection techniques for MTB in patients with TA could not corroborate these results [2, 5-7].

Patients with TA have been observed to have heightened T cell response and antibody titer to mycrobaterial 65 kDa HSP [8, 9]. This may be due to molecular mimicry or sharing of antigenic region of HSP between humans and mycobacterium tuberculosis (9). Patients of TA and TB share common inflammatory signals which can trigger Th1 and Th17 responses and granuloma formation noted in both the scenarios [10, 11].

Trehalose-6,6-dimycolate (TDM), also called as mycobacterial cord factor, is a major glycolipid present in the cell wall of mycobacterium tuberculosis. It is a potent virulence factor, which protects TB bacilli from macrophage mediated killing and inhibits effective antigen presentation [12]. Administration of TDM in mice leads to granuloma formation [13], accompanied by a cascade of pro-inflammatory cytokine production that includes TNF-α, IL-6, IL-1β, IFN-γ and IL-12 as well as some chemokines [14].

Glycolipid trehalose-6,6-dibehenate (TDB), is less toxic synthetic analogue of the TDM. It evokes similar inflammatory and granulomatous response as TDM. Both TDM and TDB bind to their receptors macrophage inducible Ca2+-dependent lectin (MINCLE ;CLEC4E) and MCL (CLEC4D) leading to the induction of Th1 and Th17 immune response via Syk - Card9 - Bcl10 - Malt1 signalling pathways [15-17].

In this study, we revisited the link of TB with TA by studying TDB-MINCLE signalling pathways in PBMCs of Asian Indian patients with TA.

## 2 PATIENTS AND METHODS

### 2.1 Participants

Patients fulfilling 1990 American College of Rheumatology criteria for Takaysu arteritis with angiographic type V disease were recruited. Age and sex matched healthy volunteers without known history of any autoimmune disease were included as controls [7]. Patients and controls were recruited after getting informed written consent from the participants. This study was approved by the Institutional Review Board (IRB), and performed in accordance with the ethical standards laid down in the 1964 Declaration of Helsinki and its later amendments.

### 2.2 Stimulation of PBMC with TDB

PBMC was separated from blood by density gradient centrifugation using Ficoll-Paque^TM^ Plus (1033378, GE Healthcare) and centrifuged at 400 g for 25 min. The buffy coat obtained was taken out and washed with sterile PBS twice. PBMC was suspended in Roswell Park Memorial Institute (RPMI) 1640 medium supplemented with 10% fetal bovine serum (Gibco, Thermo fischer ScientiifcInc, USA). PBMC of each subject was cultured at 10^6^ cells/ml in 24 well plates (Costar, Coring NY, USA) with TDB (Invivogen, CA, USA) at concentrations of 5 and 50µg/ml. The previous studies on rodents and *in-vitro* models used concentration ranging from 5 to 50 ug/ml of TDB [11, 18]. In parallel, PBMCs cultured without the addition of TDB acted as baseline control. PBMCs were cultured for 48 hrs in CO2 incubator (Forma, Thermo Scientific, USA). At the end of 48 hours, the cells and supernatants were separated and stored at −80°C till the gene expression study by RT-PCR was performed.

### 2.3 Measurement of Cytokines

IL-6, TNF-α and IL-17 were measured in supernatant by commercially available sandwich ELISA kits (R&D systems, USA) in accordance with the manufacturer’s instructions.

### 2.4 Quantification of Gene Expression

RNA was extracted from the cells using TRI reagent as per the manufacturer’s recommended protocol. RNA was quantitated by nanodrop 2000 (Thermoscientific). One microgram of RNA was used for cDNA conversion using ProtoScript First Strand cDNA synthesis kit (E6300S, New England Biolabs Inc).

Quantification of gene expression by real time PCR analysis was done in StepOnePlus™ Real-Time PCR Systems (Applied Biosystems). PCR reactions were performed using 10 μl of 1X VeriQuest SYBR Green qPCR master mix (75600, USB, AffymetrixInc, USA), 0.5 μl of 250 nM of forward primer, 0.5 μl of reverse primer, 2 μl of five times diluted cDNA and 7 μl of sterile water. The PCR conditions for individual gene expressions were optimized by gradient PCR and melting curve analysis was performed. PCR amplicons were verified by electrophoresis on 2% agarose gel, followed by visualization on a gel documentation system (AlphaImager® HP, ProteinSimple, CA, USA). We tested two housekeeping genes, Glyceraldehyde 3-phosphate Dehydrogenase (GAPDH) and human acidic ribosomal protein P0 (RPL0) for normalization of gene expression and chose RPL0 for final analysis, which has early Crossing Points (Cp) value as compared to GAPDH. All cDNA samples were then amplified for the genes of interest as well as the housekeeping gene. Table **1** depicts the list of genes studied and their primers. Cycle Threshold (Ct) or Crossing Point (Cp) values obtained from triplicate runs were analysed for quantifying target gene expression relative to the housekeeping gene using the 2−ΔCp method.

### 2.5 Statistical Analysis

Cytokine levels and quantified gene expression were compared between unstimulated and TDB stimulated PBMCs using Wilcoxon signed rank test. While, unpaired t test with Welch correction or Mann Whitney test based on normality of distribution was performed for the comparisons of cytokine levels in supernatant as well as for cytokine gene expression levels between the patients with TA and healthy controls. The cytokine levels in supernatant shown in Tables (**2** & **3**) are expressed as median (range). Statistical analysis was performed using Graphpad Prism software (Version 5; GraphPad Software, Inc, USA). p values less than 0.05 were considered as statistically significant.

## 3 RESULTS

Twenty two patients with TA and 21 healthy controls were recruited for study. Demographic characteristics for TA are shown in Table **4**. Median age of the controls was 31 (24-45) years, and male: female ratio was 1: 3.2.

**Fig. (1):**
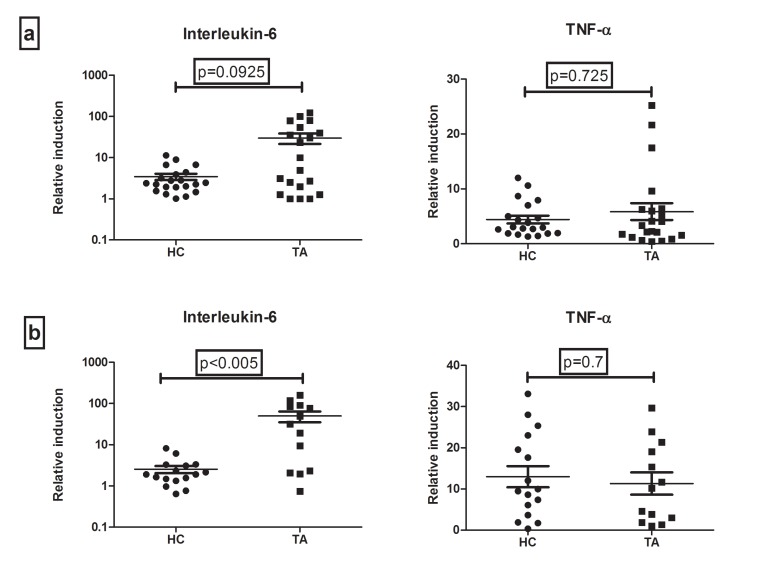
Relative Induction (fold change) of IL-6 and TNF-α levels (a) TDB 5 (TDB5 stimulated / unstimulated baseline) depicted in the upper 2 scatterplots (b) TDB 50 (TDB50 stimulated / unstimulated baseline) depicted in the lower 2 scatterplots. Values expressed as mean ± standard deviation.

Cytokine levels in the supernatant of PBMCs cultured with or without TDB (concentration of 5µg/ml and 50µg/ml) were measured (Tables **3** and **4** respectively). There was no difference in the baseline secretion of cytokines in controls and cases. However, the relative induction (TDB stimulated/ unstimulated control) of IL-6 after stimulation with 5 and 50µg/ml of TDB, was higher in cases as compared to controls. Also, the higher IL-6 induction was significant only when stimulation was done by 50µg/ml of TDB (Fig. **1**). IL-6 level induced by TDB was 31 (2.22-88) fold in TA patients as compared to healthy controls [1.9 (1.3 – 3.2)]; p<0.005. However, there was no difference in the relative induction of TNF-α secretion after stimulation with both the concentrations of TDB between TA and healthy controls. IL-17 levels were undetectable in all subjects with or without TDB stimulation. IL-6 and TNF-α protein levels did not differ between patients currently on treatment and treatment naïve patients.

MINCLE expression was significantly higher in TA as compared to healthy controls [1.03 (0.623-1.346) vs 0.43(0.36-0.57); p= 0.0111] on TDB stimulation. Relative induction of gene expressions for IL-6, IL-8, TNFα, IFN-γ and BCL-10 after TDB stimulation was not significantly different between TA and healthy controls except MINCLE as mentioned above (Table **5**).

## 4 DISCUSSION

We explored the link between tuberculosis and pathogenesis of TA by studying the recall response to mycobacterial antigen analogue TDB in PBMCs of Asian Indian patients with TA. We demonstrated that the stimulation of PBMCs with TDB led to up-regulation of its receptor MINCLE and induced increased secretion of IL-6 in cultured PBMCs of TA cases as compared to healthy controls.

The significantly increased induction of IL-6 by TDB was observed only when the cells were stimulated at TDB concentration of 50 microgram/ml and not at 5 microgram/ml. Previous studies on other diseases have also observed a concentration dependent inflammatory immune response to TDB stimulation [11]. The response in terms of TNF-α secretion also showed a similar trend, but it was not statistically significant (Table **5**). This could be because of late induction of TNF-a secretion as compared to quicker secretion of IL-6 [11]. Our culture period for 48 hours in the present study may not be long enough to induce this response.

The high expression of MINCLE had been observed in PBMCs of patients with various rheumatic diseases such as rheumatoid arthritis and Bechet’s disease. It has also been demonstrated in renal tissue of patients with lupus nephritis [19-21]. In fact, infliximab treatment in Bechet’s disease results in attenuated expression of MINCLE [20]. Lipopolysaccharide and pro-inflammatory cytokines are inducers of MINCLE expression. In the present study, the basal expression of MINCLE did not differ between TA and healthy controls, but the TDB induced expression was higher as discussed above.

TDB bound to MINCLE receptor induces transcription of pro-inflammatory cytokines through Syk-Card9-Bcl10-Malt1 signalling pathway [11]. However, in the present study, there was no difference in the relative expression of Bcl-10 among cases and controls. The mRNA expression of IL-6, IL-8, TNF-α and IFN-γ also did not differ among cases and controls in spite of stimulation with TDB. Relative mRNA expression of IL-8 was numerically higher in TA as compared to healthy controls, but it did not reach statistical significance, though there was a trend (p=0.071). IL-8 and IL-6 are early inflammatory mediators, expressed as well as secreted by PBMCs upon TDB stimulation as early as 24 hours post stimulation and the effect lasts up to 72 hrs [11, 22]. We measured gene expression at 48hrs, by which time, most of the transcribed RNAs may have been translated to proteins; thereby, it can explain the increased level of IL-6 in culture supernatants of patients as compared to controls.

Major limitation of this study was failure to get information regarding the past exposure to TB or positive tuberculin skin test (TST) and/or Quantiferon gamma test in all study participants. Hence it is not clearly known whether the previous exposure to TB in TA patients resulted in quick and increased pro-inflammatory immune response by their PBMCs on re-exposure to TDB. However, the same was true for controls as well and this could have nullified that cofounding effect to a large extent. The direct role of TDB in pathogenesis of TA could not be established in this study; therefore, a study to explore this pathway as well as to reproduce our results is needed by other centres to conclude the link between TA and TB.

## CONCLUSION

PBMCs of Asian Indian patients with TA showed higher MINCLE expression and IL-6 secretion as compared to healthy controls upon TDB stimulation.

## Figures and Tables

**Table 1:** Primers used for quantitation of select gene expressions.

***Gene***	***Froward Sequence (5′→3′)***	***Reverse Sequence (5′→3’)***	***Annealing Temperature (°C)***
*IL-6*	CATTTGTGGTTGGGTCAGG	AGTGAGGAACAAGCCAGAGC	54
*IL-8*	GAATGGGTTTGCTAGAATGTGATA	CAGACTAGGGTTGCCAGATTTAAC	52
*TNF-α*	CCTGCCCCAATCCCTTTATT	CCCTAAGCCCCCAATTCTCT	52
*IFN-γ*	TCGGTAACTGACTTGAATGTCCA	TCCTTTTTCGCTTCCCTGTTTT	54
*CLEC4E* (Mincle)	CTACCGAGTCCGTGTCTACCA	CATCCAAGTGCCAGCTAAGAG	59
*BCL10*	ATGGAGCCACGAACAACCTCT	TCGTGCTGGATTCTCCTTCTG	56
*RPLP0*	GCTTCCTGGAGGGTGTCC	GGACTCGTTTGTACCCGTTG	55
*GAPDH*	GGTGGTCTCCTCTGACTTCAACA	AGCTTGACAAAGTGGTCGTTGAG	58

IL – interleukin, TNF – Tumour necrotic factor, IFN- interferon

**Table 2:** Clinical Details of Patients with TA.

***Parameter***	***n=22***
Median age in years (range)	27 (21-32.5)
Gender (Male: Female)	4:18
Median duration of symptoms in months (range)	8 (1-60)
Median age of onset in years (range)	26.5 (15-49)
Median ESR in mm/1^st^ hour (range)	32 (15.75-51.25)
Median CRP in mg/dl (range)	6.8 (3.4-22.03)
Median ITAS (range)	7 (4-14.25)
Median DEI.Tak (range)	7.5 (4-13.25)
Medications Used	n (%)
Treatment naïve	14 (63.64%)
Second line Immunosuppression ((MMF =6) and Immunomodulation (MTX=1) along with steroids (n=7)	7 (31.82%)
Biological DMARDs	Nil

ESR – erythrocyte sedimentation rate, CRP- C reactive protein, ITAS – Indian Takayasu activity score, DEITAK - DISEASE EXTENT INDEX FOR TAKAYASU ARTERITIS, MMF - mycophenolate mofetil, MTX- methotrexate

**Table 3:** Cytokines levels secreted by unstimulated and TDB (5µg/ml) stimulated PBMCs of patients with TA and healthy controls.

***ELISA (pg/ml)***	***Unstimulated Control*** ***Median (range)***	***TDB Stimulated PBMC Supernatant Levels (5µg/ml)***	***p Value***
IL-6_HC	789(77.73-1834)	2052(452.2-4067)	< 0.0001
IL-6_TA	158(41-1948)	2313(554.5-7368)	0.0007
TNF-α_HC	20(7.5-33.5)	67(25-103)	< 0.0001
TNF-α_TA	15(7.25-36.5)	52.5(31.25-102)	0.0037

HC – healthy control, TA – Takayasu arteritis

**Table 4:** Cytokines levels secreted by unstimulated and TDB (50µg/ml) stimulated PBMCs of patients with TA and healthy controls.

***ELISA (pg/ml)***	***Unstimulated Control*** ***Median (range)***	***TDB Stimulated PBMC Supernatant Levels (50µg/ml)***	***p Value***
IL-6 HC	1236(246-1836)	2192(613.2-3492)	0.0057
IL-6 TA	129.4(50.51-1100)	5096(996.8-15061)	0.0012
TNF-α HC	23.5(10.25-42.25)	268(35-563)	0.0010
TNF-α TA	12.5(7.25-22.75)	205(33.25-463.3)	0.0017

**Table 5:** Relative Induction (fold change) of Gene expression by TDB50 stimulated / unstimulated baseline ratio in TA and healthy controls.

**Gene**	**HC (n=8)**	**TA(n=9)**	**p Value**
*IL-6*	1.03(0.58-1.45)	2.1(0.77-6.76)	0.1520
*IL-8*	0.87(0.47-1.87)	4.72(0.82-6.791)	0.071*
*TNF-α*	1.21(0.69-2.11)	0.95(0.86-2.023)	0.9182
*IFN-γ*	0.98(0.8-1.616)	0.96(0.35-2.31)	1.000
*CLEC4E*	0.43(0.36-0.57)	1.03(0.623-1.346)	**0.0111**
*BCL10*	0.33(0.28-1.62)	0.97(0.77-1.243)	0.2991

*Trend towards significance
